# Corneal subbasal nerve plexus reinnervation and stromal cell morphology with different cap thicknesses in small incision lenticule extraction

**DOI:** 10.1186/s40662-024-00381-6

**Published:** 2024-04-08

**Authors:** Yanzheng Song, Shijing Deng, Xiaotong Lyv, Yushan Xu, Fengju Zhang, Ning Guo

**Affiliations:** Beijing Ophthalmology and Visual Sciences Key Lab, Beijing Tongren Eye Center, Beijing Tongren Hospital, Capital Medical University, Beijing, 100730 China

**Keywords:** ACCMetrics, Automated analysis, Cap thickness, In vivo corneal confocal microscopy, Myopia, Refractive surgery, Small incision lenticule extraction, Subbasal nerve plexus

## Abstract

**Purpose:**

The corneal cap thickness is a vital parameter designed in small incision lenticule extraction (SMILE). The purpose was to investigate the changes in corneal subbasal nerve plexus (SNP) and stromal cells with different cap thicknesses and evaluate the optimized design for the surgery.

**Methods:**

In this prospective, comparative, non-randomized study, a total of 108 eyes of 54 patients who underwent SMILE were allocated into three groups with different corneal cap thicknesses (110 μm, 120 μm or 130 μm group). The SNP and stromal cell morphological changes obtained from in vivo corneal confocal microscopy (IVCCM) along with their refractive outcomes were collected at 1 week, 1 month, 3 months and 6 months postoperatively. One-way analysis of variance (ANOVA) was used to compare the parameters among the three groups.

**Results:**

The SNPs in the three groups all decreased after surgery and revealed a gradual increasing trend during the 6-month follow-up. The values of the quantitative nerve metrics were significantly lower in the 110 μm group than in the 120 μm and 130 μm groups, especially at 1 week postoperatively. No difference was detected between the 120 μm and 130 μm groups at any time point. Both Langerhans cells and keratocytes were activated after surgery, and the activation was alleviated during the follow-up.

**Conclusions:**

The SMILE surgeries with 110 μm, 120 μm or 130 μm cap thickness design achieved good efficacy, safety, accuracy and stability for moderate to high myopic correction while the thicker corneal cap was more beneficial for corneal nerve regeneration.

## Background

In the past decade, refractive corneal surgery has become a popular and highly specialized area with multiple techniques developed rapidly. The novel procedure of keratorefractive surgery small incision lenticule extraction (SMILE) has been widely adopted to correct refractive errors such as myopia or myopic astigmatism since it was first introduced in 2011. Given the advantages of minimal invasiveness, rapid recovery, stable biomechanics, and precise predictability [1], refractive surgeons currently prefer to use this procedure to improve vision.

Anatomically, most corneal nerves are derived from the ophthalmic branch of the 5th cranial nerve (trigeminal nerve) and sympathetic nerve. The nerve plexus moves toward the central cornea, enters the anterior stroma in a radial fashion, pierces Bowman’s layer verticality, and ends in the epithelium. The network of nerve fibers generated between Bowman’s layer and basal epithelial cells is called the subbasal nerve plexus (SNP) [2]. The characteristics of the SNP are of great value in evaluating the ocular surface-related diseases and surgeries. Corneal nerves express biologically active neuropeptides in the tear film. In other words, changes to the corneal nerve are reflected in the changes to tear film proteins [3]. Therefore, the corneal parameters of SNP have been applied to evaluate the response to treatment in dry eye disease [4]. Some of the corneal nerve fibers are cut off in keratorefractive surgery, and alterations of the SNP can be used to compare different surgical techniques [5].

The surgical design of the corneal cap thickness in SMILE surgery is an important issue. The range of the cap thickness can be designed to be 100 to 160 μm [6, 7]. To our knowledge, a cap thickness of 110 to 130 μm is commonly used for myopic correction clinically [8], whereas the optimal choice of cap thickness is not agreed around the world. Although Wu [8] reported that 120 μm is recommended as a standard parameter in China, Ganesh [7] reported a 110 μm design as routine with no special considerations in India. Some influence of the cap thickness design have been investigated and discussed, such as corneal curvature, biomechanical properties [9, 10], tissue remodeling, wound healing [11]. However, few studies have evaluated the neurological and morphological changes of the cornea [12], which may have a potential effect on the clinical refractive outcome.

In this study, we aim to investigate the changes in corneal nerve fibers and the morphological characteristics of stromal cells after SMILE with different cap thicknesses. It is hoped that through the comparison and evaluation of the characteristics of corneal tissue in patients treated with different cap thickness designs, we can obtain an optimized design for rationalization of the surgery.

## Methods

### Study design and participants

This was a prospective, comparative, non-randomized study. Patients who were willing to undergo keratorefractive surgery with a stable moderate to high myopia for a minimum period of 2 years were included in the study. Exclusion criteria included a central corneal thickness less than 480 μm, abnormal cornea (keratoconus or other corneal ectasias, edema, interstitial or neurotrophic keratitis), or other ocular disease. Diabetes was also excluded, which was known to be associated with a deficit in subbasal nerve parameters [13] and may cause some possible undesirable confounding effects in this study. Contact lens wearers were asked to discontinue lens wear for at least 2 weeks prior to examinations.

Participants underwent a comprehensive ophthalmic examination before surgery, including uncorrected distance visual acuity (UDVA), corrected distance visual acuity (CDVA) [logarithm of the minimum angle of resolution (logMAR) scale], manifest refraction spherical equivalent (MRSE) and central corneal thickness (CCT). The examination using in vivo corneal confocal microscopy (IVCCM) was done preoperatively and at 1 week and 1, 3, and 6 months postoperatively.

The Ethical Committee of the Beijing Tongren Hospital Review Board approved the study protocol (ChiTRC-TOC-17013765). The study was conducted in accordance with the principles of the Declaration of Helsinki. Prior to any procedure, written informed consent was obtained from each participant after the study had been explained to them.

### Surgical procedures

A single experienced surgeon (F.Z.) designed all the treatment parameters of the SMILE surgeries, including cap thicknesses of 110 μm, 120 μm or 130 μm, and performed the surgeries using the VisuMax femtosecond laser system (Carl Zeiss Meditec AG, Jena, Germany). The surgeon choosed different cap thickness based on the CCT and refractive correction target. The basic design principle was to ensure that the calculated postoperative residual stromal bed thickness (RST) was no less than 280 μm. Different nomograms were applied for dioptric adjustment of spherical equivalent correction according to the cap thickness [14]. All other surgical parameters were the same between patients. The optical zone diameter was 6.5 mm, cap diameter was 7.6 mm and the transition zone diameter was set as 0.1 mm. The surgical procedures and the management of the perioperative period were carried out following standard clinical protocols [15].

### In vivo corneal confocal microscopy

IVCCM was performed using a Heidelberg Retina Tomograph III laser scanning confocal microscope with the Rostock Cornea Module (HRT III RCM; Heidelberg Engineering GmbH, Heidelberg, Berlin, Germany) by a single experienced technician (S.D.), who was blinded to the surgical design of cap thickness. Approximately 100 digital images from the basal epithelium to endothelial cells of the cornea were recorded from both eyes. Each image covered an area of 400 μm × 400 μm with a transverse optical resolution of 2 μm and a longitudinal optical resolution of 4 μm. Representative images of good quality were chosen from the basal epithelium, SNP, surgically extracted lenticule plane (approximately 10 μm close to the surgical plane anteriorly and posteriorly), posterior stromal, and endothelial cell layers in the central cornea for image analysis. Among those, five to eight images of SNPs were selected, and quantitative nerve metrics were analyzed automatically with the software ACCMetrics (ACCMetrics version 2.0, Center for Imaging Sciences, University of Manchester, Manchester, UK). The following seven parameters were obtained: corneal nerve fiber density (CNFD), the number of main nerve fibers per mm^2^ (no./mm^2^); corneal nerve branch density (CNBD), the total number of nerve primary branch points on the main nerve fibers per mm^2^ (no./mm^2^); corneal nerve fiber length (CNFL), the total length of main nerve fibers and branches per mm^2^ (mm/mm^2^); corneal nerve fiber total branch density (CTBD), the total number of branch points per mm^2^ (no./mm^2^); corneal nerve fiber area (CNFA), the total nerve fiber area per mm^2^ (mm/mm^2^); corneal nerve fiber width (CNFW), the average nerve fiber width per mm^2^ (mm/mm^2^); and corneal nerve fractal dimension (CNFrD), the ratio of the change in detail to the change in scale.

### Statistical analysis

The statistical analysis was performed using SPSS (version 24.0). Descriptive data are shown as the mean ± standard deviation (SD). The Kolmogorov‒Smirnov test was used to check for normality of distribution. Data transformation methods such as square root, inverse, or log (base 10 or natural log) transformation were used when the data did not conform to the standard normal distribution. If the assumption of normality was violated, the nonparametric Kruskal‒Wallis test was used. One-way analysis of variance (ANOVA) was used to compare the continuous variables of corneal nerve parameters and refractive outcomes among three groups of cap-thickness design. Repeated measures ANOVA was used to compare the variables at different time points postoperatively. Multiple comparisons between groups were carried out after ANOVA, and the Student–Newman–Keuls (SNK) method was applied for post hoc testing. The *P* value threshold was set at 0.05 in all cases.

## Results

### Baseline demographic and ophthalmic examination data before refractive surgery

From the original cohort, we included a total of 108 eyes of 54 patients (17 males and 37 females) who underwent SMILE at Beijing Tongren Eye Center between August 2020 and November 2021 in the study. The mean age was 25.7 ± 4.23 years old (ranging from 18 to 33 years). Of these, 14 patients were in the 110 μm group, 23 patients were in the 120 μm group, and 17 patients were in the 130 μm group. More than 90% of subjects (49 out of 54 patients) completed all 6-month refractive examinations in our study. In addition, 14 patients, 16 of 23 patients and 14 of 17 patients in the three groups, respectively, completed all IVCCM follow-up examinations, which accounted for 81% of the subjects in this study. We followed up with the remaining patients by phone. They all reported good visual quality after surgery but were unable to complete the examinations due to personal reasons such as conflicts in schedule and availability.

The baseline characteristics of demographic and ophthalmic examination data before the SMILE surgery are shown as the mean ± SD in Table 1. As shown in the data, the CCT of the 110 μm group was significantly thinner than that of the 120 μm and 130 μm groups. It was consistent with clinical reality—for patients with thin corneas, the surgeon tended to choose a thinner corneal flap design to preserve a thicker corneal RST. In addition, there was no statistical difference in baseline data such as preoperative sphere, MRSE, lenticule thickness and RST between the 110 μm and the 130 μm groups after post hoc testing, which facilitates our analysis of refractive outcomes and corneal nerve parameters between the two groups.

**Table 1:** Baseline demographic and ophthalmic examination data before refractive surgery (mean ± SD)

Parameter	Groups			*P* value
	110 μm	120 μm	130 μm	
Age (years)	23.89 ± 3.93	27.15 ± 3.25	25.22 ± 5.25	0.064
CDVA (logMAR)	− 0.07 ± 0.03	− 0.07 ± 0.02	− 0.07 ± 0.02	0.865
Preoperative sphere (D)	− 4.69 ± 0.99	− 5.27 ± 0.92	− 4.46 ± 0.62	0.000*^a^
Preoperative cylinder (D)	− 0.36 ± 0.40	− 0.68 ± 0.61	− 0.52 ± 0.59	0.065
MRSE (D)	− 4.88 ± 0.90	− 5.62 ± 0.87	− 4.72 ± 0.64	0.004*^a^
CCT (μm)	538.69 ± 17.32	552.98 ± 16.63	559.79 ± 13.70	0.000*^b^
LT (μm)	109.33 ± 13.07	122.25 ± 13.72	108.25 ± 11.73	0.007*^a^
RST (μm)	319.36 ± 12.82	310.91 ± 18.70	322.21 ± 18.01	0.012*^a^

*D* = diopters; *CDVA* = corrected distance visual acuity; *MRSE* = manifest refraction spherical equivalent; *CCT* = central corneal thickness; *LT* = lenticule thickness; *RST* = residual stromal bed thickness

*Indicate statistical significance (*P* < 0.05)

^a^The data in 120 μm group is significantly different from the other two groups, whereas there was no difference between 110 μm group and 130 μm group

^b^The data in 110 μm group is significantly different from the other two groups, whereas there was no difference between 120 μm group and 130 μm group

### Refractive outcomes

All surgeries were completed without intraoperative complications. No vision-threatening complications occurred throughout the 6-month follow-up period. There was no significant difference in MRSE among the three groups at any time point postoperatively (*P* > 0.05; Table 2). All subjects in our study had obtained UDVA better than 20/25 at any time point, and the UDVA was slightly better in the 110 μm group at 1 week, 1 month and 6 months postoperatively. The efficacy index (EI, ratio between mean postoperative UDVA and mean preoperative CDVA) was 1.71, 1.00, and 1.29, respectively. The safety index (SI, ratio between mean CDVA at 6 months postoperatively and preoperatively) was 1.71, 1.29 and 1.43, respectively. Our data revealed that 89%, 87% and 91% of eyes were within ± 0.5 D of attempted correction in the 110 μm, 120 μm and 130 μm groups, respectively. All eyes in these three groups achieved within ± 1.0 D of attempted correction.

**Table 2:** Refractive outcomes at follow-ups

Parameter	Groups			*P* value
	110 μm	120 μm	130 μm	
UDVA (logMAR)				
1w postop	− 0.10 ± 0.06	− 0.04 ± 0.10	− 0.04 ± 0.08	0.004*
1m postop	− 0.11 ± 0.07	− 0.07 ± 0.06	− 0.07 ± 0.07	0.030*
3m postop	− 0.09 ± 0.06	− 0.10 ± 0.06	− 0.07 ± 0.06	0.230
6m postop	− 0.12 ± 0.06	− 0.07 ± 0.07	− 0.09 ± 0.05	0.001*
MRSE (D)				
1w postop	− 0.15 ± 0.52	0.01 ± 0.36	− 0.11 ± 0.36	0.125
1m postop	0.07 ± 0.40	0.17 ± 0.31	− 0.01 ± 0.33	0.147
3m postop	0.18 ± 0.42	0.15 ± 0.38	0.07 ± 0.30	0.495
6m postop	0.08 ± 0.27	0.08 ± 0.35	0.05 ± 0.30	0.994

*D* = diopters; *UDVA* = uncorrected distance visual acuity; *logMAR* = logarithm of the minimum angle of resolution; *MRSE* = manifest refraction spherical equivalent; *m* = month; *w* = week; *postop* = postoperative

*Indicate statistical significance (*P* < 0.05)

### Corneal SNP measurement

**Fig. 1:**
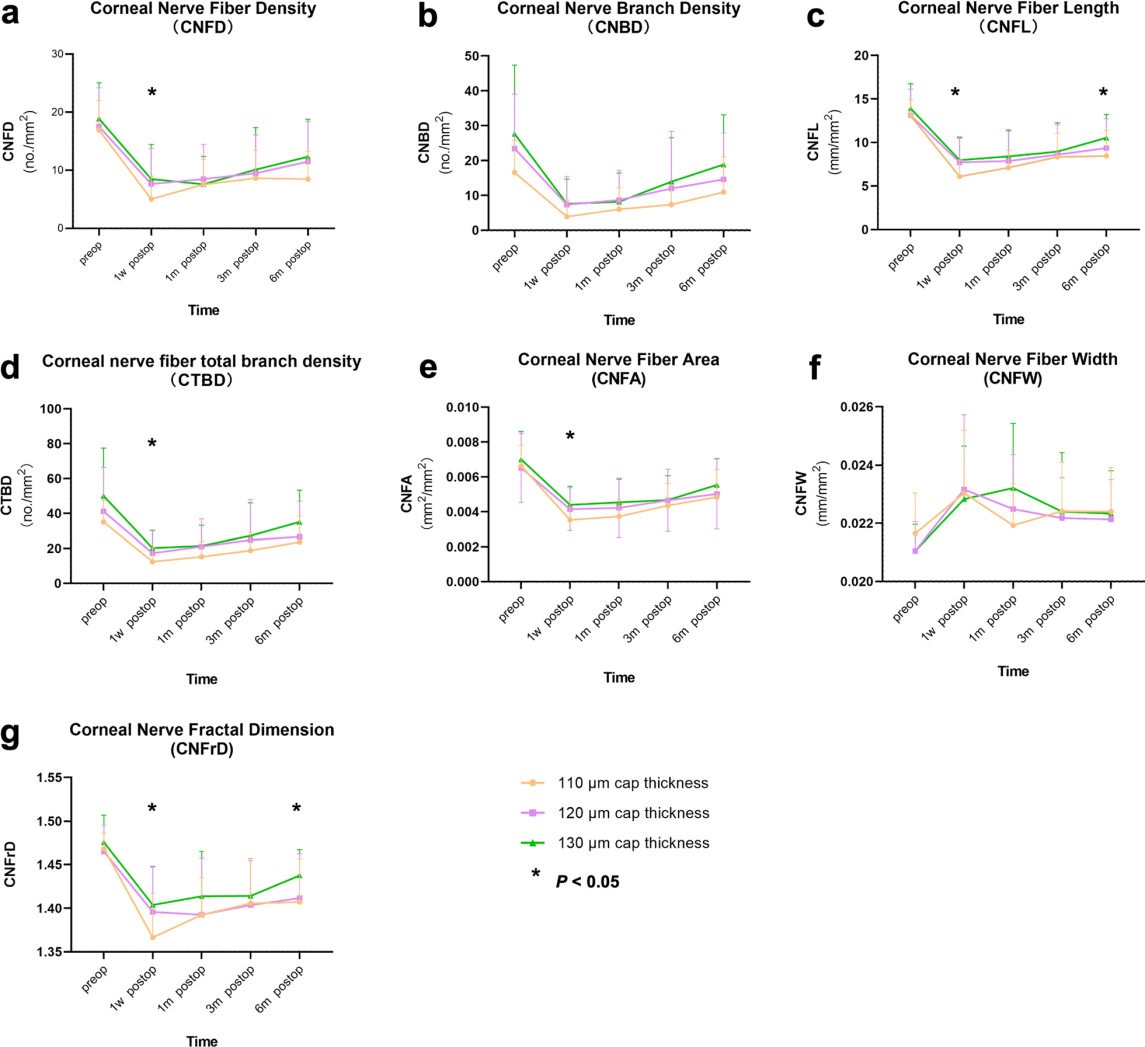
The alteration trend of seven corneal SNP parameters preoperatively and postoperatively during the 6-month follow-up period. **a** Corneal nerve fiber density (CNFD); **b** corneal nerve branch density (CNBD); **c** corneal nerve fiber length (CNFL); **d** corneal nerve fiber total branch density (CTBD); **e** corneal nerve fiber area (CNFA); **f** corneal nerve fiber width (CNFW); **g** corneal nerve fractal dimension (CNFrD). CNFD (**a**), CNFL (**c**), CTBD (**d**), CNFA (**e**), and CNFrD (**g**) in the 110 μm group were significantly lower than those in the other two groups at 1 week postoperatively. CNFL (**c**) and CNFrD (**g**) in the 110 μm group were significantly lower than those in the other two groups at 6 months postoperatively

The corneal nerve parameters obtained from IVCCM are shown in Table 3 and Fig. 1. There was no significant difference in any corneal nerve parameter among the three groups preoperatively. It is easy to tell from Table 3 and Fig. 1 that parameters such as CNFD, CNBD, CNFL, CTBD, CNFA and CNFrD decreased after surgery with an increasing trend at the time point of 1 month. The differences among the time points of 1 month, 3 months and 6 months within each group were significant. The differences in CNFD, CNFL, CTBD, CNFA and CNFrD among the three groups at 1 week postoperatively were significant. Multiple comparisons showed that the data of the 110 μm group were significantly decreased compared with those of the other two groups. All corneal SNP parameters at 1 month and 3 months postoperatively were comparable among the three groups. At 6 months, CNFL and CNFrD were significantly lower in the 110 μm group than in the other two groups. In addition, it can be seen from the figure that most of the metrics of the 130 μm group showed the highest value among the three groups although not to a statistically significant level.

**Table 3:** Subbasal nerve plexus (SNP) measurement with different cap-thickness design at baseline and follow-ups

Parameter	Time point					
	Preop	1w postop	1m postop	3m postop	6m postop	*P* value
CNFD (no./mm^2^)						
110 μm	16.943 ± 5.013	5.026 ± 3.967	7.537 ± 4.355	8.628 ± 4.810	8.471 ± 4.798	0.001*
120 μm	17.532 ± 6.689	7.634 ± 6.131	8.476 ± 5.985	9.492 ± 6.603	11.490 ± 6.884	0.026*
130 μm	18.882 ± 6.178	8.495 ± 5.969	7.599 ± 4.759	10.121 ± 7.206	12.328 ± 6.465	0.000*
*P*	0.467	0.017*	0.853	0.925	0.052	
CNBD (no./mm^2^)						
110 μm	16.548 ± 9.258	3.896 ± 4.733	6.040 ± 6.056	7.367 ± 5.547	10.943 ± 10.110	0.001*
120 μm	23.430 ± 15.620	7.354 ± 8.075	8.690 ± 8.500	11.968 ± 16.389	14.620 ± 13.283	0.009*
130 μm	27.637 ± 19.751	7.585 ± 7.102	8.180 ± 8.272	13.932 ± 12.643	18.836 ± 14.288	0.000*
*P*	0.061	0.095	0.593	0.391	0.107	
CNFL (mm/mm^2^)						
110 μm	13.078 ± 1.868	6.092 ± 1.881	7.109 ± 1.991	8.347 ± 2.726	8.460 ± 2.892	0.000*
120 μm	13.112 ± 3.030	7.720 ± 2.952	7.878 ± 3.406	8.604 ± 3.431	9.356 ± 3.364	0.016*
130 μm	13.904 ± 2.839	7.983 ± 2.538	8.405 ± 3.035	8.963 ± 3.296	10.534 ± 2.684	0.000*
*P*	0.244	0.012*	0.250	0.877	0.040*	
CTBD (no./mm^2^)						
110 μm	35.092 ± 12.384	12.409 ± 7.792	15.212 ± 8.670	18.829 ± 10.151	23.645 ± 14.870	0.002*
120 μm	41.209 ± 25.266	17.309 ± 12.706	21.045 ± 15.806	24.757 ± 23.124	26.657 ± 20.403	0.077
130 μm	50.008 ± 27.473	20.205 ± 10.081	21.459 ± 11.841	27.236 ± 18.805	35.159 ± 18.220	0.000*
*P*	0.071	0.014*	0.367	0.840	0.055	
CNFA (mm/mm^2^)						
110 μm	0.007 ± 0.001	0.004 ± 0.001	0.004 ± 0.001	0.004 ± 0.001	0.005 ± 0.002	0.000*
120 μm	0.007 ± 0.002	0.004 ± 0.001	0.004 ± 0.002	0.005 ± 0.002	0.005 ± 0.002	0.007*
130 μm	0.007 ± 0.002	0.004 ± 0.001	0.005 ± 0.001	0.005 ± 0.001	0.006 ± 0.002	0.000*
*P*	0.186	0.017*	0.097	0.668	0.292	
CNFW (mm/mm^2^)						
110 μm	0.021 ± 0.001	0.023 ± 0.002	0.022 ± 0.001	0.022 ± 0.002	0.022 ± 0.002	0.360
120 μm	0.021 ± 0.001	0.023 ± 0.003	0.022 ± 0.002	0.022 ± 0.001	0.022 ± 0.001	0.000*
130 μm	0.021 ± 0.001	0.023 ± 0.002	0.023 ± 0.002	0.022 ± 0.002	0.022 ± 0.001	0.032*
*P*	0.076	0.856	0.084	0.903	0.762	
CNFrD (ratio scale)						
110 μm	1.468 ± 0.017	1.366 ± 0.051	1.392 ± 0.042	1.406 ± 0.051	1.407 ± 0.049	0.000*
120 μm	1.465 ± 0.030	1.396 ± 0.053	1.393 ± 0.065	1.404 ± 0.051	1.412 ± 0.051	0.210
130 μm	1.476 ± 0.031	1.404 ± 0.044	1.414 ± 0.051	1.414 ± 0.043	1.438 ± 0.030	0.002*
*P*	0.260	0.010*	0.191	0.673	0.025*	

*CNFD* = corneal nerve fiber density; *CNBD* = corneal nerve branch density; *CNFL* = corneal nerve fiber length; *CTBD* = corneal nerve fiber total branch density; *CNFA* = corneal nerve fiber area; *CNFW* = corneal nerve fiber width; *CNFrD* = corneal nerve fractal dimension

The *P* values in the right column show the statistical difference among different time points postoperatively, and the *P* values at the bottom row of each category represent the statistical difference among three groups

*Indicate statistical significance (*P* < 0.05)

### Morphological changes in corneal nerve fibers and keratocytes

In addition to quantitively assessing the corneal SNP, the morphological changes of corneal nerve fibers and interstromal cells located close to the depth of the cap thickness were also observed by IVCCM.

**Fig. 2:**
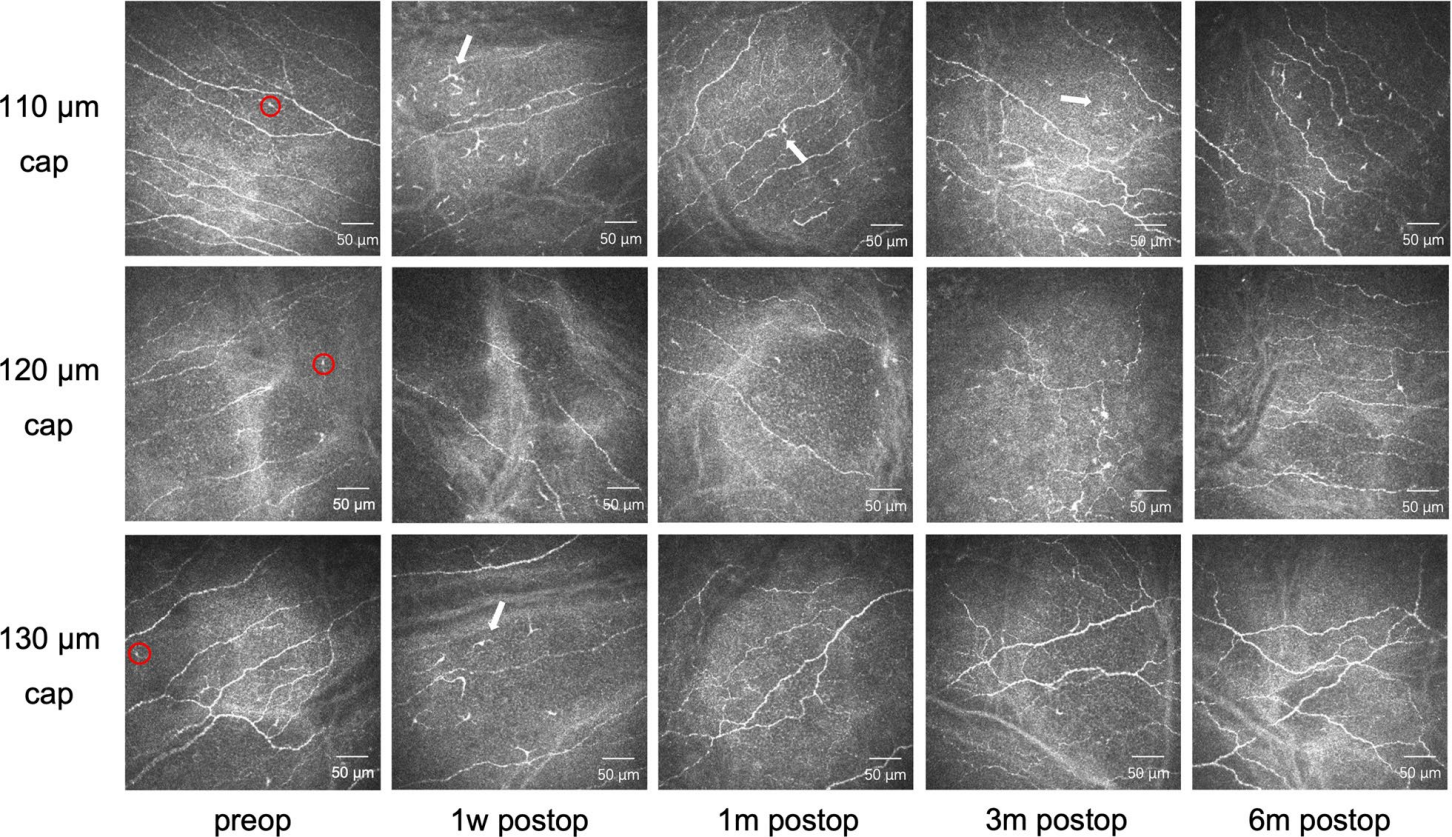
Representative images of subbasal nerve plexus (SNP) recovery in three different groups. The corneal SNP with some inactivated Langerhans cells (red circle) were detectable in all eyes preoperatively. The density and length of corneal nerve fibers were decreased after surgery at 1 week postoperatively, and a significant decrease was observed in the 110 μm group. The nerve fibers revealed an increasing trend at 1 month postoperatively, while the corneal nerve tortuosity increased 3 months after the surgery. Some dendritic cells (white arrow) were detected after surgery. The set of five images in each group showing alterations of the SNP during the 6-month follow-up after the procedure were taken from the same individual. Field size: 400 μm × 400 μm. m, month; w, week; preop, preoperative; postop, postoperative

**Fig. 3:**
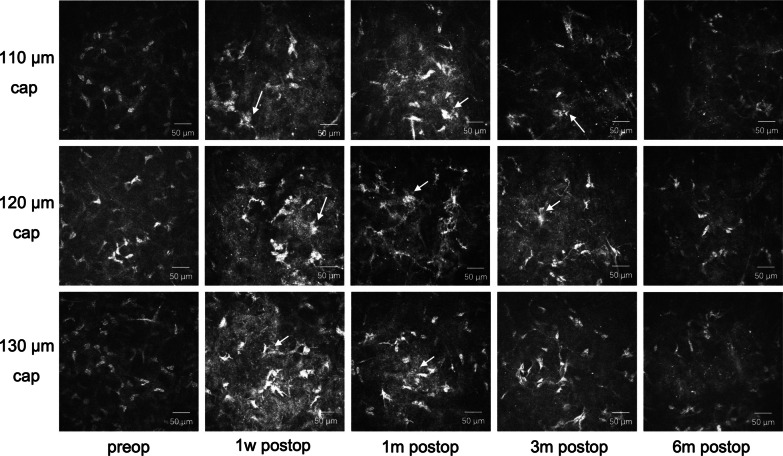
Representative images of extracted lenticule plane in three different groups. The activation of keratocytes was alleviated time-dependently. Some light-scattering particles were observed (white arrow), which may be due to keratocyte necrosis and apoptosis. The set of five images in each group showing alterations of the keratocytes during the 6-month follow-up after the procedure were taken from the same individual. Field size: 400 μm × 400 μm. m, month; w, week; preop, preoperative; postop, postoperative

Representative images of corneal SNP preoperative distribution and recovery after surgery in the three groups are shown in Fig. 2. The corneal SNP with some inactivated Langerhans cells were detectable in all eyes preoperatively. The SNP densities were decreased in the three groups at all postoperative follow-up visits for 6 months, with the greatest reduction at 1 week, whereas a gradual increasing trend was observed throughout the follow-up period. Some active Langerhans cells, which were also recognized as dendritic cells, were detected in the layer of the SNP after surgery. High reflectivity was detected at the extracted lenticule plane. The keratocytes in the surgical interface (Fig. 3) were activated, which was revealed as a distinctive hyperreflective state. Some light-scattering particles were observed, which may be due to keratocyte necrosis and apoptosis and can be recognized as a mark of the extracted lenticule plane. The alleviation of activation was observed 1 month after surgery and the keratocyte nuclei became more quiescent over time during the 6-month follow-up visit. Unlike the preoperative appearances of corneal nerve fibers, which were parallel and straight, the nerve fibers appeared tortuous at the 3-month and 6-month follow-up visits, which were less straight and thinner with more branches and networks.

## Discussion

It has been widely accepted and confirmed that the SMILE procedure has promising refractive outcomes. The SI and EI scores are important indices when evaluating refractive surgery. It is accepted to be safe when the SI score is equal to or greater than one. Similarly, when the EI score is equal to or greater than one, it indicates that the patient is satisfied with the procedure. All SMILE procedures with different cap thicknesses in our study showed satisfying visual outcomes. The greatest reduction in corneal SNP was observed at postoperative week 1, and an increasing trend was observed throughout the follow-up, which is consistent with previous studies [12, 16].

IVCCM is becoming an indispensable ophthalmic imaging technique that obtains high-resolution visualization of corneal SNPs and keratocytes [17]. The software ACCMetrics by the University of Manchester offers quantified analyses of SNPs, and the nerve metrics have been proved to be reproducible and repeatable [13, 18–21]. This methodology has been applied in longitudinal studies and helps gain a full scope of information on the time-dependent trends of corneal regenerative capacity after SMILE surgery [5]. The parameters CNFL, CNFD, CNBD, and CTBD are primary markers when comparing the alterations in corneal nerve fibers in several studies.

The nerve fibers are partly removed in SMILE surgery, so it is easy to understand from our observation that the corneal nerve parameters were reduced after surgery. The decrease in CNBD and CTBD indicates damage in the distal branches, while the alterations in CNFD indicate that changes occur in more proximal nerves [18]. High CNFrD values correspond to a healthier and evenly distributed complex nerve fiber structure, while lower values point to fewer distorted nerve fibers, potentially reflecting abnormality [22]. We hypothesized that it is the depth of the created corneal cap that causes the slight differences in the corneal nerve fiber parameters among the three groups during the 6-month recovery course. In other words, different cap thicknesses may influence corneal nerve fiber damage and reinnervation. Of note, the value of CNFW was the only one out of seven parameters that did not reveal a downwards trend after surgery. The pattern of CNFW in Fig. 1 even showed an increasing upside at 1 week postoperatively although not to a statistically significant level. It is postulated that the pathological course induced by the procedure occurred instantaneously and may not significantly affect the width of corneal nerve fibers as other chronic neuropathy courses. Nevertheless, the procedure may induce nerve fiber swelling in the early period after surgery.

In addition to these quantitative results obtained from the software, morphological changes should also be noted. The postoperative changes include increased tortuosity and beading, some mature Langerhans cells and activated keratocytes. Increased tortuosity and beading represent the recovery process of the corneal fibers. Langerhans cells are antigen-presenting cells of the cornea. They usually lack dendrites as their immature form and are located mainly in the subbasal layer of the peripheral cornea. Their mature form can also be called dendritic cells, revealing an interlocking structure, and migrating from the periphery into the central cornea in some pathological states. It has been proven that there is a significant correlation between corneal nerve loss and an increase in Langerhans cell density in patients with diabetes, in which the corneal nerve is believed to be affected [23]. It is hypothesized that the increasing presence of dendritic cells may be an indication of the ocular surface immune system being alert and ready to rapidly activate, which may occur after surgery [24]. The increased intensity of stromal reflectivity and the activated keratocytes at the lenticule extraction plane can usually be seen as changes to the underlying stroma because of the laser stimulation or the process steps of lenticule dissection and extraction [25]. During the 6-month period of follow-up visit, the interface stromal activity subsided in all three groups. Along with the SNP changes we mentioned above, these results indicate the gradual healing response and recovery process postoperatively.

Corneal cap thickness is a vital parameter during SMILE surgery. Many comparisons have been made with different SMILE cap thickness designs, involving refractive outcomes, biomechanics, corneal sensitivity, and dry eye conditions after surgery [6, 8, 16, 26, 27]. However, comparisons involving corneal nerve density, reinnervation and stromal cell morphology have rarely been reported. The design of cap thickness may affect corneal characteristics and consequently the outcomes of SMILE surgery from at least the following four aspects: (i) corneal nerve fiber reinnervation; (ii) corneal curvature; (iii) corneal biomechanic characteristics; and (iv) procedure design if retreatment is needed.

First, in our study, a more superficial cap depth may possibly interfere with more nerve plexus. Although others found that the thickness of the cap creation did not affect clinical outcomes postoperatively [10, 11], a thinner cap may be associated with dry eye postoperatively because of the corneal sensitivity [16] due to neurogenic factor [7]. Interestingly, the conclusion is inconsistent when compared with the flap thickness design in laser in situ keratomileusis (LASIK) surgery. The underlying mechanisms may be explained as follows: the deeper the corneal flap is cut, the more severe the damage to nerves of the main branch [28]. However, Yang et al. [12] evaluated corneal nerve destruction following SMILE in patients who had a corneal cap thickness of 100 μm or 120 μm and found no difference in the density and number of nerve fibers under the basement membrane between these groups at 1 week following surgery. The discrepancy in conclusions between Yang’s study and ours may be related to the different methods used for SNP quantification assessment.

Second, some researchers found that corneal cap thickness affects the change in the curvature of the anterior surface [9]. Liu’s findings suggested that modification of the corneal shape and the change in anterior surface curvature would be easier using a 110 μm cap thickness [29]. Other studies [30] claimed that a thicker corneal cap may not correct refractive errors effectively because of less flattening of the anterior curvature through their ex vivo experiments. This is partly consistent with our results of refractive outcomes and another previous study [15]. Although the UDVA was slightly better in the 110 μm group, especially in the early period after surgery, we should note that the UDVA result was easily affected by the examination environment subjectively, and there was no statistically significant difference in MRSE. Güell suggested that MRSE correction should be increased by 3% for every 10 μm increase in corneal cap thickness [31], which was also applied in this current study. We recommend that the nomogram design be modified to overcome possible changes in laser energy. In addition, the epithelium thickness profile changes following SMILE for moderate to high myopia correction could not be ignored. The epithelium remodeling response may have an impact on the refractive outcome especially in higher degree of myopia [32]. Hence, the influence of cap thickness on epithelium remodeling warrant further study.

Third, it is believed that different corneal cap thicknesses may affect postoperative corneal biomechanics. Our previous study showed that the 130 μm group had the most stable biomechanics [15]. This finding is consistent with the corneal biomechanical characteristics anatomically and theoretically. The anterior stroma contributes more to the biomechanical stability of the cornea than the posterior stroma, and a thicker corneal cap could spare more anterior stroma and minimize changes in the biomechanics after SMILE [9, 33] although Liang [34] concluded that there was no significant difference in biomechanics among corneal caps with different thicknesses.

Finally, the primary corneal cap thickness plays an important role in SMILE retreatment. If refractive regression occurs, a thicker cap may leave more space within or above the cap, increasing the possibility for enhancement, such as excimer laser ablation, thin flap LASIK and secondary SMILE [35]. Although it is theoretically a potential advantage, more clinical trials with large sample sizes are warranted to validate the statement [6, 31].

There were some limitations in our current study. First, the ideal study would follow the principle of randomization. However, it is difficult to achieve randomized cap thickness and matched lenticule thicknesses because we must save the RST within a safety threshold to ensure the corneal biomechanism postoperatively. Therefore, for some patients with thin corneas, the surgeon may choose a thinner cap thickness design rather than a randomized cap thickness in clinical practice. Some contralateral eye studies that adopted a randomized study design pertaining to cap thickness demonstrated that mean CCT should be more than 550 μm preoperatively to ensure the safety of the surgery [9, 11, 33]. Second, it would be a better statistical design that data collected and analyzed from one eye per patient. However, the cornea-contactable method of IVCCM would limit the number of subjects. Some complained of discomfort due to the examination which is partly the reason why the sample size decreased during the 6-month follow-up. Further studies utilizing a randomized study design and larger sample size will be needed to extend the findings of this study.

## Conclusions

We found that all SMILE surgeries with 110 μm, 120 μm and 130 μm corneal cap designs in our study achieved good efficacy, safety, accuracy, and stability. However, the decrease in SNP was severe in the 110 μm group at 1 week after surgery. Considering the nerve reinnervation process, a thicker cap-thickness design may be preferred for correcting refractive errors with moderate to high myopia and astigmatism with sufficient residual stromal bed thickness ensured for safety.

## Data Availability

The datasets used and analyzed during the current study are available from the corresponding author on reasonable request.

## Abbreviations

SMILE: Small incision lenticule extraction
SNP: Subbasal nerve plexus
UDVA: Uncorrected distance visual acuity
IVCCM: In vivo corneal confocal microscopy
CNFD: Corneal nerve fiber density
CNBD: Corneal nerve branch density
CNFL: Corneal nerve fiber length
CTBD: Corneal nerve fiber total branch density
CNFA: Corneal nerve fiber area
CNFW: Corneal nerve fiber width
CNFrD: Corneal nerve fractal dimension
ANOVA: Analysis of variance
CCT: Central corneal thickness
MRSE: Manifest refraction spherical equivalent
CDVA: Corrected distance visual acuity
logMAR: Logarithm of the minimum angle of resolution
LT: Lenticule thickness
RST: Residual stromal bed thickness
EI: Efficacy index
SI: Safety index
